# 

**Published:** 1938

**Authors:** 


### CONTENTS.

| | Page |
|---|---|
| Psychological Factors in Disease. By T. M. Ling, B.M., B.Ch., M.R.C.P. | 101 |
| Cerebral Malformations and their Clinical Consequences. By R. J. A. Berry, M.D., F.R.C.S., F.R.S.E. | 111 |
| The Use of Protamine Insulin. By J. A. Nixon, C.M.G., M.D., F.R.C.P. | 121 |
| Reviews of Books | 132 |
| Editorial Notes | 140 |
| Obituary: C. A. H. Gee, M.B., Ch.B. | 145 |
| Meetings of Societies | 146 |
| The Medical Library of the University of Bristol | 146 |
| Publications Received | 148 |
| Local Medical Notes | 149 |

